# Analysis of axial shortening induced by orthokeratology lenses and its mechanical mechanisms

**DOI:** 10.1371/journal.pone.0323546

**Published:** 2025-05-12

**Authors:** Zhao-Yang Meng, Lin Yang, Peng Zhou

**Affiliations:** 1 Department of Ophthalmology, Beijing Friendship Hospital, Capital Medical University, Beijing, China; 2 Department of Ophthalmology, Visionly Plus Eye Hospital, Beijing, China; 3 Department of Ophthalmology, Parkway Gleneagles Medical and Surgical Center, Shanghai, China; 4 Department of Ophthalmology, Parkway Hong Qiao Medical Center, Shanghai, China; The Ohio State University, UNITED STATES OF AMERICA

## Abstract

**Purpose:**

This study aims to examine the short-term axial shortening effects of orthokeratology (ortho-K) lenses and investigate their mechanical mechanisms.

**Methods:**

We conducted a retrospective analysis on 80 myopic children, aged 8–18, who wore ortho-K lenses for one week. Axial lengths were measured pre- and post-treatment using AL-Scan Optical Biometer. We developed a finite element model of the eye using ABAQUS software to explore mechanical changes.

**Results:**

A significant reduction in axial length was observed after one week of ortho-K lens wear, with an average decrease of 0.028 ± 0.032 mm (P = 4.02 x 10^-11^). Approximately 82.5% of participants exhibited axial length reduction. The biomechanical model indicated that ortho-K lenses exerted forces altering the tension dynamics within the ocular structure, notably decreasing tension in the posterior ciliary muscle-lens complex. This differential change in tension may account for the mechanical basis of the observed short-term reduction in axial length.

**Conclusion:**

Orthokeratology lenses induce a short-term shortening in axial length, likely due to mechanical changes in ocular tension dynamics. The finite element model suggests that these lenses decrease posterior ciliary-lens complex tension, leading to axial shortening. These findings enhance comprehension of the mechanical basis for myopia control via ortho-K treatment, highlighting potential avenues for further applied research in myopia management.

## Introduction

Myopia has emerged as a major public health concern on a global scale [1,2]. The prevalence is particularly high among middle school-aged youths in East and Southeast Asia, with rates reported between 70% and 90%,looking ahead to 2050, forecasts predict a dramatic increase, with approximately 5 billion individuals, accounting for half of the global population, expected to suffer from myopia, and 10% anticipated to have high myopia [3,4]. The severe form of myopia is associated with a heightened risk of several complications, such as cataracts, retinal detachment, glaucoma, macular atrophy, and choroidal neovascularization. These conditions pose serious threats to visual health, potentially leading to significant visual impairment and irreversible blindness [5,6]. Consequently, it is crucial to develop and implement strategies to curb the progression of myopia.

Orthokeratology has been recognized as an effective intervention for managing myopic progression [7]. This non-surgical method involves the overnight wear of specially designed gas-permeable contact lenses that temporarily reshape the cornea, thereby correcting refractive errors [8]. Numerous studies have demonstrated the efficacy of orthokeratology in not only improving uncorrected vision during the day but also in significantly reducing the rate of myopia progression when compared to traditional corrective spectacles or soft contact lenses [7,9].

Orthokeratology has garnered attention for its potential in managing myopia by influencing axial length (AL) [10,11]. Recent studies highlight the intriguing phenomenon of AL reduction in individuals using orthokeratology lenses [12]. Although long-term AL shortening in myopic patients is considered rare, a comprehensive review of orthokeratology records over a decade revealed that a notable fraction of individuals exhibited significant AL reduction, with the process predominantly occurring within the first two years of lens wear [13]. This reduction’s incidence correlated strongly with the age at onset of orthokeratology treatment [14]. Furthermore, another study examining axial variation in children demonstrated that AL can experience short-term reductions within the first month of orthokeratology [15]. Importantly, these early AL reductions have been proposed as potential predictors for effective long-term myopic control [16]. The synthesis of findings from these studies underscores the potential for orthokeratology to not only provide temporary refractive corrections but also influence underlying ocular growth patterns [17].

The mechanism by which orthokeratology lenses lead to axial shortening remains a topic of investigation. Previous studies hypothesize that the peripheral defocus induced by the altered corneal shape might play a critical role in mitigating ocular elongation, the anatomical change associated with myopic progression. However, our previous research suggests that the ciliary muscle and the crystalline lens significantly contribute to axial elongation in myopic eyes [18]. We hypothesize that the short-term axial changes induced by orthokeratology lenses are primarily due to mechanical effects exerted by the orthokeratology. Therefore, we designed this experiment to explore the mechanical mechanisms through which orthokeratology lenses influence axial length changes.

In this study, we have compiled clinical cases to analyze the ultra-short-term (one week) changes in axial length induced by orthokeratology, focusing particularly on whether axial shortening occurs. Subsequently, we utilized the ABAQUS software to construct a finite element model of the eyeball, aiming to investigate the mechanical mechanisms by which orthokeratology lenses may lead to axial shortening.

## Materials and methods

### Study framework and participant recruitment

This retrospective analysis was performed at three ophthalmic institutions: Parkway Gleneagles Medical and Surgical Center (PG), Shenton Health Hong Qiao Medical Center (SH), and Visionly Plus Eye Hospital (VP). While PG and SH, situated in Shanghai, are globally acclaimed for medical excellence, providing services to both local Chinese and international patients, VP focuses on serving the Chinese demographic exclusively. Data collection was conducted from January to September 2024, followed by data analysis. The criteria set for inclusion were: (i) participants aged 8–18 years, (ii) a cycloplegic spherical equivalent (SE) less than –1.0 diopters in the more affected eye, (iii) no significant ocular health issues, (iv) parental or guardian consent, and (v) no history of orthokeratology or low-dose atropine eye drop use.

### Ethics statement

The study adhered to the ethical standards laid out by the Declaration of Helsinki. Ethical clearance was obtained from the Parkway Gleneagles Medical and Surgical Center’s Ethics Committee and Institutional Review Board (IRB) (approval no. 202312). Due to its retrospective nature with anonymous data, the study was granted a waiver of informed consent by the IRB.

### Ortho-K lens application and monitoring

Experienced practitioners undertook the fitting of ortho-K lenses following the manufacturer’s instructions and after thorough assessments. Follow-up visits were scheduled one week post initiation of lens wear. During these visits, ophthalmologists assessed axial length, ortho-K lens hygiene, and conducted anterior segment examinations to identify any signs of corneal epithelium staining, corneal edema, or conjunctival congestion.

### Axial length measurement technique

The methodology for determining axial length mirrored that of our preceding investigations [19]. To ensure uniformity, this technique was standardized across all participating ophthalmic centers. Ophthalmologists, trained for consistency, performed slit lamp examinations using a slit lamp lens (Digital Wide Field, Volk, USA) to inspect both the anterior and posterior eye segments. Axial measurements were conducted utilizing the AL-Scan Optical Biometer (Nidek, Japan) prior to dilation. Although measurements were taken for both eyes, this study focused on data from the right eye of each participant.

### Biomechanical modeling

The approach to constructing the Finite Element Model was consistent with that of our previous studies [18]. A 2-D finite element biomechanical model of the eye was created using ABAQUS 2020 software (Dassault Systèmes SIMULIA Corp.). As lens alterations were beyond the study’s scope, a compound “ciliary muscle-lens complex” was modeled. Experimental conditions included an axial length of 22.78mm, a 5.73mm distance from the cornea to the lens’s posterior surface, a 17.05mm gap from the lens’s posterior surface to the eye’s posterior pole, and an eye wall thickness of 0.67mm. The source files for this biomechanical model can be found in the supplementary data (S1 and S2) and are compatible with the ABAQUS software.

### Statistical methodology

The requisite sample size was determined using an online Sample Size Calculator (https://homepage.univie.ac.at/robin.ristl/samplesize.php). The study successfully included data from 80 right eyes of 80 patients, satisfying the necessary sample size criteria. Statistical analyses were performed using the R programming language (version 4.1.3). A paired Student’s t-test was applied to compare axial lengths pre- and post-ortho-K lens application. Differences between the eyes were assessed using the Pearson correlation coefficient, with statistical significance set at P < 0.01.

## Results

### Characteristics of the study cohort

The cohort for this research comprised 80 right eyes of 80 myopic children. Among these participants, 47.50% (38) were boys, while 52.50% (42) were girls. Predominantly, the group consisted of individuals of Chinese descent, accounting for 88.75% (71), with the remaining 11.25% (9) being Caucasian. Participants were aged between 8.01 and 17.27 years, and the median age was identified as 10.05 years. Prior to the initiation of orthokeratology lens use, the mean spherical equivalent was recorded at -3.57 ± 1.22 diopter sphere (Ds), within a range from -1.50 Ds to -6.00 Ds.

### Alterations in axial length associated with orthokeratology lens use

**Fig 1:**
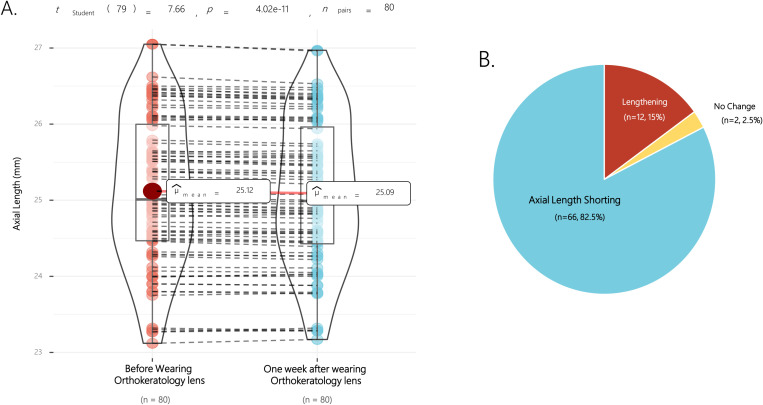
Axial length changes after one week of wearing orthokeratology lenses. 1A: The paired violin plot depicts the alteration in axial length before and after wearing orthokeratology lenses. The axial length prior to wearing orthokeratology lenses is longer by 0.028 ± 0.032mm compared to after wearing the lenses. 1B: The diagram demonstrates the distribution of cases showcasing a reduction in axial length following wearing orthokeratology lenses. A vast majority of cases (82.5%) featured a decrease in axial length after wearing orthokeratology lenses.

Upon evaluating changes in axial length attributable to Orthokeratology lens wear, a notable reduction was observed post-treatment relative to pre-treatment conditions. Fig 1A visually represents the pre- and post-Orthokeratology axial length measurements (the anonymized data set is in Supporting Information 3). Initially, the average axial length was recorded at 25.12 ± 0.98 mm (mean ± standard deviation), which subsequently decreased to 25.09 ± 0.96 mm following the use of Orthokeratology lenses. This denotes a reduction of 0.028 ± 0.032 mm compared to the baseline value. The statistical analysis of these variations was significant (paired t-test: t = 7.66, P = 4.02 x 10^-11^). No significant discrepancies in axial length were noted between the eyes prior to and following Orthokeratology intervention, as evidenced by high Pearson correlation coefficients (pre-treatment, r = 0.9591; P < 0.001, post-treatment, r = 0.9357, P < 0.001).

In terms of proportionality, an appreciable 82.5% (66 of 80 eyes) of the sample displayed a decrease in axial length following Orthokeratology lens use. Conversely, a smaller fraction, 15% (12 of 80 eyes), exhibited an increase, while the remaining 2 eyes showed no change, as depicted in Fig 1B.

### Distribution of forces exerted by orthokeratology lenses on the ocular structure

Utilizing ABAQUS software, we developed a finite element representation of the ocular anatomy. Given that our study did not involve changes to lens morphology, we have simplified the ciliary muscle and lens into an integrated “ciliary muscle-lens complex.” The thickness for each ocular component is calibrated based on a “standardized eye.” Further details regarding the ABAQUS model database for ciliary muscle contraction can be found in Supporting Information 1 accompanying this manuscript.

**Fig 2:**
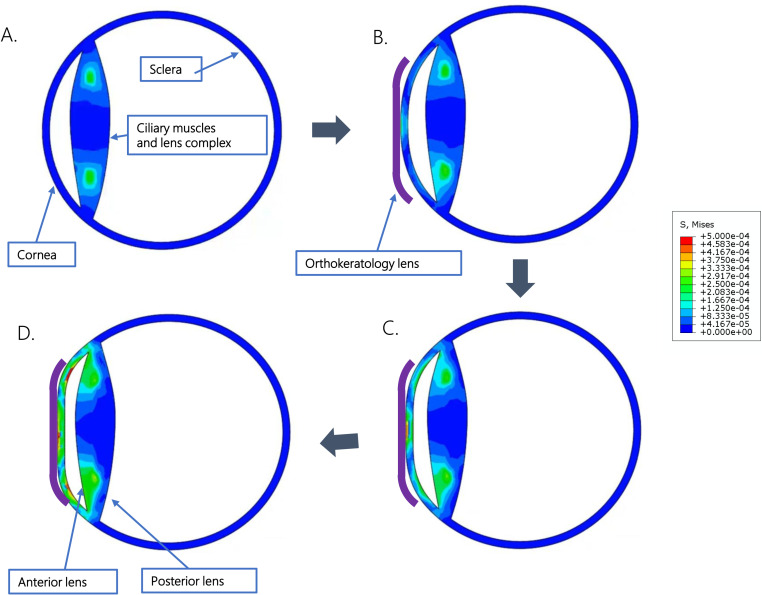
Changes in ocular stress after wearing orthokeratology lenses. The finite element model of the eyeball was constructed using ABAQUS software. The ciliary muscle and lens were simplified into a “ciliary muscle-lens complex”. The application of orthokeratology lenses induces a force distribution, with stress levels (indicated by red, yellow, and green) commencing from the corneal contact surface and propagating toward the posterior segment of the eye (A to D). It is noteworthy that there is an elevation in tension within the anterior portion of the complex, while the posterior section experiences a reduction in tension (D).

As illustrated in Fig 2, the application of orthokeratology lenses induces a force distribution, with stress levels (indicated by red, yellow, and green) commencing from the corneal contact surface and propagating toward the posterior segment of the eye. This phenomenon notably affects the tension dynamics within the ciliary muscle-lens complex. It is noteworthy that there is an elevation in tension within the anterior portion of the complex, while the posterior section experiences a reduction in tension. An animated depiction of these tension variations during the contraction of the ciliary muscle is available in Supporting Information 2 of this manuscript.

### The relationship between axial length changes induced by orthokeratology lenses and corneal thickness

Based on our previous findings indicating an association between corneal thickness and myopia progression [19], this study further examines the relationship between central corneal thickness and changes in axial length following the use of orthokeratology lenses. The results demonstrate a statistically significant correlation between these two variables (r = -0.433, P < 0.001). However, the correlation coefficient suggests that this relationship is of low magnitude.

## Discussion

This study investigates the short-term alterations in axial length following the use of orthokeratology lenses and further develops a model to explore changes in ocular tension induced by these lenses. Our findings indicate that after one week of wearing OK lenses, the axial length decreased by 0.028 ± 0.032 mm. Additionally, post-lens application, there is an increase in tension on the anterior portion of the ciliary-lenticular complex, while the posterior tension decreases. This differential change in tension may account for the mechanical basis of the observed short-term reduction in axial length.

A distinguishing feature of this study, in comparison to previous research [12,17], is its exclusive focus on changes in axial length one week after orthokeratology lens wear, categorizing it as an ultra-short-term investigation. The rationale for selecting a one-week duration is to concentrate on the mechanical causes of axial length changes, thereby minimizing the impact of other confounding factors, particularly peripheral defocus [20–22]. Previous research has indicated that wearing orthokeratology lenses can lead to changes in axial length due to various factors such as central corneal thickness, anterior chamber depth, lens thickness, and choroidal thickness [15,23]. Among these, choroidal thickness exhibits significant temporal variability and is likely influenced by peripheral defocus [24,25]. Given that changes induced by peripheral defocus tend to manifest gradually, we opted for an ultra-short-term timeframe of one week to mitigate its effects.

In this study, it was observed that after one week of orthokeratology (OK) lens wear, the axial length decreased by 0.028 mm. This finding is consistent with trends reported in previous research. Lau et al. reported a decrease in axial length of 0.026 mm after one week of OK lens wear [15]. Similarly, Gardener et al. found a reduction of 0.04 mm after one month of wear [26]. However, studies with longer observation periods generally indicate an increase in axial length. For example, Li et al. observed an axial length increase of 0.06 mm after six months of wear [27], and Swarbrick et al. noted an increase of 0.04 mm over the same period [28]. In summary, orthokeratology lenses appear to induce a short-term reduction in axial length, but there is a tendency for axial growth over longer periods of wear.

The biomechanical model developed in this study reveals intriguing changes in the tension of the ocular wall and the ciliary muscle-lens complex following orthokeratology lens wear. Initially, these lenses exert pressure on the central corneal region, and the resultant tension is transmitted posteriorly. However, due to the relatively low magnitude of this pressure, its propagation is limited, reaching only as far as the ciliary muscle. This phenomenon leads to an increase in tension within the anterior segment of the ciliary muscle.

A particularly significant finding is the reduction in stress within the posterior ciliary muscle-lens complex. This phenomenon leads to the relaxation of the ciliary muscle and a subsequent increase in lens thickness, which is consistent with previous clinical studies. One study observed that after one week of orthokeratology lens wear, the lens thickness increased by 32 μm [15]. Additionally, another study, a randomized 2-year clinical trial, found an approximate increase of 10 μm in crystalline lens thickness after 24 months of wearing the lenses [29].

The significance of the reduction in stress within the ciliary muscle-lens complex lies in its potential to induce a shortening of the axial length of the eye. Our previous research findings have demonstrated that a decrease in the stress of the posterior ciliary muscle-lens complex results in axial shortening of the eye [18]. Consequently, The model helps explain the short-term axial shortening observed with these lenses.

Furthermore, the biomechanical model developed in this study illustrates how orthokeratology lenses induce alterations in the ocular wall, accounting for findings reported in numerous previous clinical studies [30]. For instance, our model reveals that the compression exerted by orthokeratology lenses leads to an increase in corneal tension, potentially resulting in corneal thinning. This observation is consistent with prior clinical results. Studies by Lau et al. [15] and Gardner et al. [26] both demonstrated a 10 μm thinning of the cornea following one week of orthokeratology lens wear. Additionally, Li et al. [27] observed a 12 μm thinning of the cornea after one month of lens wear. Moreover, our model predicts anterior chamber shallowing due to the compression from orthokeratology lenses, an outcome corroborated by past research. For example, Lau et al. [15] identified a 54 μm shallowing of the anterior chamber following lens use. It is noteworthy that the shallowing of the anterior chamber following the use of orthokeratology lenses might also be attributed to overcorrection associated with the Jesson Factor. This overcorrection can prompt lens accommodation, thereby increasing lens thickness and subsequently causing the anterior chamber to become shallower. Collectively, these studies suggest that the alterations in the eye revealed by our biomechanical model align with clinical findings, thereby affirming the model’s reliability.

In our previous research, we developed a biomechanical model to investigate the transmission of tension through the ocular wall induced by ciliary muscle contraction, contributing to theoretical studies on myopia [18]. The current study, however, presents a novel biomechanical model that examines the effects of orthokeratology lens pressure on the tension of the ocular wall, representing an applied research focus. The Abacus source files for both models are included in the supplementary materials of these papers. We invite interested ophthalmologists to use these source files for further in-depth study. However, this is definitely not an attempt to refute previous mechanisms regarding orthokeratology’s control of myopia. On the contrary, by integrating previous theories of optical defocus, this study aims to provide a more comprehensive explanation of the mechanisms by which orthokeratology controls myopia.

Our research has certain limitations. The study cohort comprises both Chinese and Caucasian children, thus providing a more comprehensive racial representation. However, it is important to consider potential differences in ocular anatomical structures among different races, such as central corneal thickness and axial length. Due to the relatively small sample size of this study, we were unable to thoroughly investigate ethnic differences. We plan to continue our clinical observations and aim to explore the racial variations in the efficacy of orthokeratology with a larger sample size in future studies. Furthermore, while our study proposes the hypothesis of “mechanical pressure - ciliary muscle relaxation - axial shortening,” it is important to acknowledge that this is primarily a theoretical model that has not yet been empirically validated. The lack of direct experimental data supporting this relationship represents a limitation in our research. As such, future studies are necessary to gather empirical evidence through rigorous experimental or observational studies to test the proposed pathway. Moreover, our investigation has primarily clarified the mechanical principles associated with the short-term effects of orthokeratology lenses on the reduction of axial length. However, there is a limited understanding of the long-term mechanical impacts, especially over periods of one to two years or more. Therefore, it is essential to conduct future long-term studies to explore how mechanical alterations influence scleral growth and other related factors.

In conclusion, this study demonstrates that the axial length of the eye shortens following the use of orthokeratology lenses. Our biomechanical model suggests that this axial shortening may result from the relaxation of the posterior ciliary-lens complex due to the pressure exerted by the orthokeratology lenses.

## Supporting information

S1 Files The ABAQUS model database of wearing orthokeratology lenses. The files included in the attachment can be opened and executed by the ABAQUS 2020 software (Dassault Systèmes SIMULIA Corp.).
(ZIP)

S2 File The animation of tension changes after wearing orthokeratology lenses. (MP4)

S3 File Anonymized data set: the anonymized data set necessary to replicate our study. (XLSX)
